# The interplay between telomeric complex members and BCR::ABL1 oncogenic tyrosine kinase in the maintenance of telomere length in chronic myeloid leukemia

**DOI:** 10.1007/s00432-023-04662-w

**Published:** 2023-03-05

**Authors:** Anna Deręgowska, Monika Pępek, Iwona Solarska, Marcin M. Machnicki, Katarzyna Pruszczyk, Marek Dudziński, Joanna Niesiobędzka-Krężel, Ilona Seferyńska, Waldemar Sawicki, Maciej Wnuk, Tomasz Stokłosa

**Affiliations:** 1 Department of Biotechnology, Institute of Biology and Biotechnology, College of Natural Sciences, University of Rzeszow, Pigonia 1, 35-310 Rzeszow, Poland; 2 Department of Tumor Biology and Genetics, Medical University of Warsaw, Pawińskiego 7, 02-106 Warsaw, Poland; 3 Molecular Biology Laboratory, Department of Diagnostic Hematology, Institute of Hematology and Transfusion Medicine, 02-776 Warsaw, Poland; 4 Department of Hematology, Institute of Hematology and Transfusion Medicine, 02-776 Warsaw, Poland; 5 Department of Hematology, Institute of Medical Sciences, College of Medical Sciences, University of Rzeszow, Rzeszow, Poland; 6 Department of Hematology, Transplantation and Internal Medicine, University Clinical Centre, Medical University of Warsaw, 02-097 Warsaw, Poland; 7 Department of Hematology, Military Institute of Medicine-National Research Institute, 04-141 Warsaw, Poland

**Keywords:** Chronic myeloid leukemia, Leukemic stem cells, *BCR::ABL1*, Telomeres, Telomerase, Shelterin complex

## Abstract

**Purpose:**

Chronic myeloid leukemia (CML) is a myeloproliferative neoplasm characterized by recurrent genetic aberration in leukemic stem cells, namely Philadelphia chromosome caused by reciprocal translocation t(9;22)(q34;q11). In our study, we analyzed the telomeric complex expression and function in the molecular pathogenesis of CML.

**Methods:**

We employed CD34+ primary leukemic cells, comprising both leukemic stem and progenitor cell populations, isolated from peripheral blood or bone marrow of CML patients in chronic and blastic phase to analyze the telomere length and telomeric-associated proteins.

**Results:**

The reduction in telomere length during disease progression was correlated with increased expression of *BCR::ABL1* transcript and the dynamic changes were neither associated with the enzymatic activity of telomerase nor with gene copy number and expression of telomerase subunits. Increased expression of *BCR::ABL1* was positively correlated with expression of *TRF2*, *RAP1*, *TPP1*, *DKC1*, *TNKS1*, and *TNKS2* genes.

**Conclusions:**

The dynamics of telomere length changes in CD34+ CML cells is dependent on the expression level of *BCR::ABL*, which promotes the expression of certain shelterins including *RAP1* and *TRF2*, as well as *TNKS*, and *TNKS2*, and results in telomere shortening regardless of telomerase activity. Our results may allow better understanding of the mechanisms responsible for the genomic instability of leukemic cells and CML progression.

**Supplementary Information:**

The online version contains supplementary material available at 10.1007/s00432-023-04662-w.

## Introduction

Chronic myeloid leukemia (CML) is a model neoplastic disease and constitutes an excellent example of translating basic knowledge into targeted therapy and clinical benefit (Jabbour and Kantarjian 2022). CML is characterized by recurrent genetic aberration in leukemic stem cells, namely the Philadelphia chromosome caused by reciprocal translocation t(9;22)(q34;q11) that leads to the formation of *BCR::ABL1* fusion oncogene (Nowell and Hungerford 1960; Rowley 1973). The hybrid gene *BCR::ABL1* undergoes translation into chimeric protein, which exerts constitutive tyrosine kinase activity and phosphorylates target proteins to facilitate survival and expansion of leukemic stem cells (Lin-CD34 + CD38 −) and progenitor cells (CD34 + CD38 +). Thus, CML is frequently described as leukemia stem cell (LSCs)-derived, but leukemia progenitor cell (LPCs)-driven disease (Marley and Gordon 2005). Progression of CML is characterized by successive increase in amount of blast cells in blood and bone marrow and is classified by phases: chronic phase (CML-CP), accelerated phase (CML-AP), and advanced blastic phase (CML-BP) also called blast crisis (Jabbour and Kantarjian 2022). A drug resistance and/or disease progression in CML despite all major milestones constitutes a significant clinical problem in a number of patients (Perrotti et al. 2010; Skorski et al. 2012). Searching for new markers useful as potential prognostic and/or predictive factors in CML is still a challenge and the subject of many studies (Wenn et al. 2015; Niederwieser and Kröger 2022).

One of such potential prognostic markers postulated were changes in telomere length because it was shown that telomere length was shorter in CML cells as compared to age-matched healthy individuals or *BCR::ABL1*-negative T lymphocytes from the same patients (Brümmendorf et al. 2000). Furthermore, telomere shortening was accelerated as the disease progressed from CML-CP to CML-BP, with a shortening rate approximately 10× higher than in normal controls (Iwama et al. 1997; Drummond et al. 2004; Wang et al. 2014; Bouillon et al. 2018). Unraveling the mechanisms and the role of telomeric complex in *BCR::ABL1*-mediated genomic instability may contribute to the development of new strategies for preventing or counteracting resistance phenotype and malignant progression of the disease. This may create new and unique therapeutic opportunities, as shown in acute myeloid leukemia as an effective strategy to eradicate leukemia stem cells (Bruedigam et al. 2014; Mascarenhas et al. 2021).

Telomere maintenance in malignant cells is associated with reactivation of telomerase, and/or the telomerase-independent alternative lengthening of telomeres (ALT) (Gao and Pickett 2022). It is well known that telomere maintenance is regulated not only by telomerase, but also by Telomeric Repeat-containing RNA (TERRA) (Silva et al. 2021) and various telomere-associated proteins, such as the shelterin complex composed of 6 proteins: telomeric repeat-binding factors 1 and 2 (TRF1 and TRF2), protection of telomeres (POT1), TRF2-interacting protein 1 (RAP1), TRF1-interacting nuclear factor 2 (TINF2), and TIN2-interacting protein 1 (TPP1), as well as other telomeric-associated proteins (telomerase associated protein (TEP1) and tankyrase). TRF1 and TRF2 along with TINF2, which prevents TRF1/TRF2 degradation by tankyrase, form the central hub of the shelterin complex that protects telomeres from being recognized as DNA double-strand breaks thereby avoiding inappropriate end-joining and DNA repair. Thus, TRF1 and TRF2, and PinX1 (TRF-interacting telomerase inhibitor 1) act as negative regulators of telomere length, while telomerase and tankyrase are positive regulators (Hockemeyer and Collins 2015; de Lange 2018). Telomere length in normal and malignant cells is regulated by a delicate balance between these factors (Augereau et al. 2011). Interestingly, so far most studies on CML cells focused on the analysis of the dependence of telomere length on telomerase activity (Brümmendorf et al. 2000; Drummond et al. 2004, 2005; Wenn et al. 2015; Bouillon et al. 2018), but characterization of the telomeric complex and telomere maintenance, including shelterin complex, in CD34+ CML cells has never been analyzed in detail.

In this work, we have investigated the effects of *BCR::ABL1*-mediated changes on expression of shelterin complex, *TERT*, telomerase RNA component (*TERC)* and dyskerin pseudouridine synthase 1 (*DKC1)* in CML cells including CD34+ primary cells isolated from peripheral blood or bone marrow of CML patients at different stages of disease. We showed for the first time that *BCR::ABL1* promoted telomere shortening independent of telomerase activity by overexpression of some shelterin genes.

## Materials and methods

### Patient samples and cell lines

Blood or bone marrow samples were obtained from 76 CML patients (55 in CML-CP and 21 in CML-BP) with confirmed Philadelphia chromosome and *BCR::ABL1* translocation and from 4 healthy volunteers. A peripheral blood mononuclear cells were isolated from CML patients or healthy blood donors using Histopaque 1077 and Histopaque 1119 (Sigma-Aldrich, Saint Louis, MO, USA), then progenitor CML CD34 + cells were selected using magnetic beads system EasySep CD34 + Positive Selection Kit (StemCell Technologies, Vancouver, Canada) according to the manufacturer’s recommendation. CD34 + cells were maintained in Iscove’s modified Dulbecco’s medium (IMDM; Lonza, Basel, Switzerland) supplemented with 10% fetal bovine serum (FBS) and growth factors: 2 ng/ml hrIL-3, 10 ng/ml hrGM-CSF and 2 ng/ml hrSCF (PeproTech, Cranbury, NJ, USA) at 37 °C in a humidified atmosphere of 5% CO_2_.

32D clone 3, interleukin (IL)-3-dependent cell line, and their *BCR::ABL1*-transformed counterparts were described before (Koptyra et al. 2006). The cells were cultured in Roswell Park Memorial Institute 1640 medium (RPMI, Lonza, Basel, Switzerland) with 10% FBS and 4 ng/ml mrIL-3 (PeproTech, Cranbury, NJ, USA) at 37 °C in a humidified atmosphere of 5% CO_2_.

### RNA isolation and cDNA synthesis

Total RNA was extracted from peripheral blood samples using QIAamp RNA Blood Mini Kit (Qiagen, Hilden, Germany) according to the manufacturer's protocol. Reverse transcription was done with Transcriptor First Strand cDNA Synthesis Kit (Roche, Basel, Switzerland) following the manufacturer's protocol.

### Real-time* qPCR* analysis

The levels of the mRNA expression of *BCR::ABL1*, *TRF1*, *TRF2*, *RAP1*, *POT1*, *TINF2*, *TPP1*, *TNKS1*, *TNKS2*, *DKC1*, *TERC*, and *TERT* were measured using LightCycler^®^ 480 Probes Master and Universal Probe Library (UPL) (Roche, Basel, Switzerland**)**. The reactions were performed using LightCycler^®^ 480 instrument (Roche, Basel, Switzerland) in a final volume of 10 µl. *B2M* and *GUSB* were used as reference genes (Supplementary Table 1).

### Fluorescence in situ hybridization (FISH)

FISH was performed using probes for: *BCR/ABL1* t(9;22) fusion (KBI-10005, Kreatech, Amsterdam, Netherlands), *TERT* (5p15) (KBI-40113, Kreatech, Amsterdam, Netherlands), or *TERC* (3q26)/3q11 (KBI-10110, Kreatech, Amsterdam, Netherlands). For FISH experiments, the procedure used has been described elsewhere (Deregowska et al. 2020).

### Telomere length analysis and telomerase activity

Average telomere length was analyzed by Southern blotting analysis and at the level of the single cell by FISH. The genomic DNA was extracted using the QIAamp DNA Blood Mini Kit (Qiagen, Hilden, Germany) and the Wizard^®^ Genomic DNA Purification Kit (Promega, Madison, WI, USA) for patients’ samples and murine cells, respectively, according to the manufacturer’s protocols. Terminal restriction fragments (TRF) length was measured by Southern blot method, using TeloTAGGG telomere length assay kit (Roche, Basel, Switzerland) following the manufacturer's protocol as described previously (Wnuk et al. 2014). In single cells, metaphase chromosomes were prepared as described elsewhere (Deregowska et al. 2020), and then Q-FISH was performed with the Telomere PNA FISH kit/Cy3 (Dako, Glostrup, Denmark) according to the protocol provided by the manufacturer’s recommendation.

Telomerase activity (TA) was measured with a TeloTAGGG Telomerase PCR ELISA kit (Roche, Basel, Switzerland) according to the manufacturer’s instructions.

### Statistical analysis

Statistical analysis was performed in GraphPad Prism 6.07 software (GraphPad Software, Inc., La Jolla, CA, USA) by *t* test with the Mann–Whitney test and by one-way ANOVA and with the Tukey’s multiple comparison test. The correlation analysis was performed using linear correlation (Pearson *r*) test.

## Results

### Decreased telomere length correlates with increased *BCR::ABL1* gene expression in CML cells

**Fig. 1:**
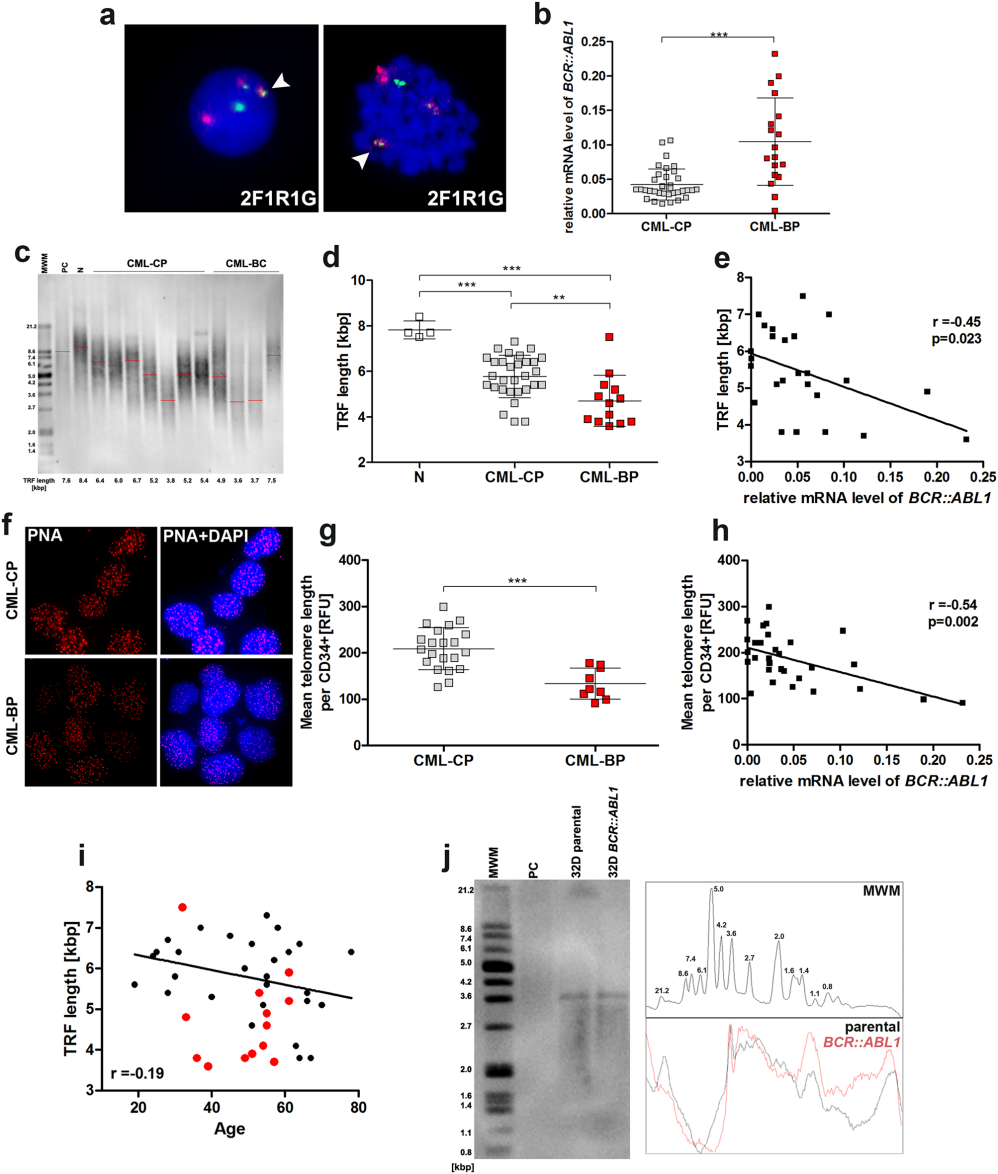
Changes in telomere length depending on CML phase and *BCR::ABL1* expression level in CD34+ primary leukemic cells isolated from patients at different stages of disease (CML-CP and CML-BP). **a** Interphase and metaphase FISH images with typical *BCR::ABL1* gene rearrangements observed among the study groups using LSI *BCR/ABL1* probe localized on chr.22q11.2 and chr.9q34, respectively (signal pattern 2F1R1G). Sequences flanking the *ABL1* (9q34) gene are direct labeled with PlatinumBright™550. Sequences flanking the *BCR* (22q11) gene are direct-labeled PlatinumBright™495. **b** The *BCR::ABL1* expression level normalized to *B2M* and *GUSB* measured with qPCR (non-parametric Student’s *t* test and Mann–Whitney post-test) ***p < 0.001, *n* = 71. **c** Representative images from Southern blot analysis. Mean TRF length (kbp) is shown in the legend of the particular lanes. Lanes: 1 and 15 DIG-molecular weight marker (MWM), [0.8–21.2], 2 and 15 control DNA [7.6 ± 0.2] (PC). **d** Comparison of telomere length measured by TRF method in leukocytes isolated from healthy donors (N) and patients at different stages of disease: CML-CP, CML-BP expressed as kbp, (ANOVA and Tukey’s a posteriori test). ***p < 0.001, *p < 0.05, *n* = 47. **e** Correlation between the level of mRNA *BCR::ABL1* expression and telomere length measured by TRF method (Pearson’s (*r*) *p* < 0.05). **f** Representative microphotographs of Q-FISH for CML CD34 + primary cells isolated from CML-CP, CML-BP patients. Telomere PNA FISH kit/Cy3 (Dako) was used for telomere labeling. Nuclei were counterstained by DAPI. **g** Means of telomere area (pixel per spot) per CML CD34 + primary cell isolated from CML-CP, CML-BP patients measured by Q-FISH with PNA technique, (non-parametric student *t* test and Mann–Whitney post-test). **p < 0.01, *n* = 30. **h** Correlation between level of mRNA *BCR::ABL1* expression and telomere length measured by Q-FISH method (Pearson’s (*r*) *p* < 0.05). **i** Correlation between age and telomere length. (Pearson’s (*r*) *p* < 0.05), *n* = 43. Red dots—CML-BP cells, black dots—CML-CP cells. **j** Southern blot analysis of telomere length in murine myeloid 32D clone 3: parental and transfected with the *BCR::ABL1* gene, MWM—molecular weight marker, PC—positive control. Densitometric profile was performed to correspond to bands of DNA marker using ImageJ with gel analysis module

With the aim of assessing the prognostic importance of changes in the length of telomeres in leukemic leukocytes of patients with CML and discovering the mechanisms responsible for the dynamism of changes in telomere length, we have conducted research on leukocytes including CD34+ primary leukemic cells, comprising both leukemic stem and progenitor populations isolated from patients at the CML-CP and CML-BP stages of the disease. The cells of all patients with the presence of the *BCR::ABL1* gene (Fig. 1a) were characterized by *BCR::ABL1* transcriptional activity (Fig. 1b). Comparative analysis of the expression of the fusion gene *BCR::ABL1* by qPCR within the group showed a 2.5 fold increase in *BCR::ABL1* expression in the cells of CML-BP patients in relation to CML-CP patients. However, it should be noted that a larger variability in the expression profile of *BCR::ABL1*, in general, was observed in the CML-BP group rather than in cells isolated from CML-CP patients.

We decided to verify the model of telomere biology in CML proposed by Brümmendorf et al. on the basis of two techniques, TRF method and Q-FISH analysis, and correlation analysis between obtained results and the level of expression of the *BCR::ABL1* gene (Brümmendorf et al. 2000). Additionally, we attempted to explain the phenomenon of changes in telomere length in patients with CML, as observed by Brümmendorf and other researchers. TRF and Q-FISH analysis confirmed that the mean length of telomeres in the CML-BP phase was significantly shorter in comparison with CML-CP phase telomeres (Fig. 1c–g). Moreover, the correlation analysis showed that the dynamics of this process may be related to the level of *BCR::ABL1* expression, that is, cells with higher *BCR::ABL1* expression had significantly shorter telomeres (Fig. 1e, h). The analysis of the relationship between age of CML patients and mean length of telomeres has shown that telomere length slightly decreased with age (Fig. 1i).

To investigate the effect of BCR::ABL1 tyrosine kinase on telomere length, 32D cells expressing *BCR::ABL1,* and the parental cell line 32D clone 3 not expressing *BCR::ABL1* were compared (Fig. 1j). Telomere lengths were quantified using Southern blot, and the results showed heterogenization of telomere length in *BCR::ABL1-*expressing and non-expressing cells.

### Changes in the levels of *TERT* and *DKC1* expression do not affect a global telomerase activity in CML cells

**Fig. 2:**
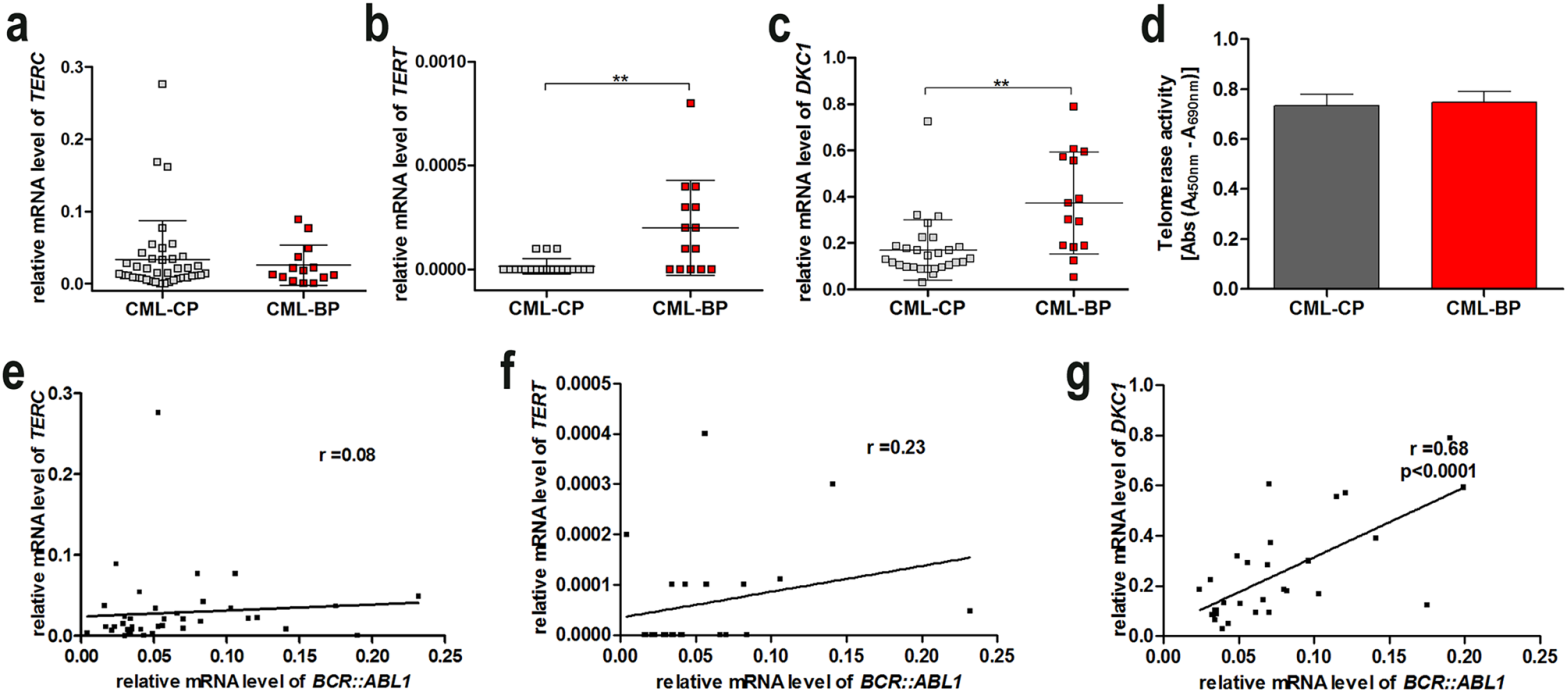
*BCR::ABL1*-mediated effect on *TERC, TERT,* and *DKC1* expression of telomerase components during CML progression. **a**–**c** The *TERC* (*n* = 54), *TERT* (*n* = 33), and *DKC1* (*n* = 42) expression levels normalized to *B2M* and *GUSB* measured with qPCR **p < 0.01(non-parametric Student’s *t* test and Mann–Whitney post-test). **d** PCR ELISA measurement of telomerase activity, *n* = 10 (non-parametric student *t* test and Mann–Whitney post-test). **e**–**g** Correlation between *BCR::ABL1* and *TERC*, *TERT* or *DKC1* expression level, respectively (Pearson’s (*r*)) *p* < 0.05

Analysis of the number of copies of *TERT* and *TERC* genes by FISH did not show statistically relevant differences between CML-BP CD34+ and CML-CP CD34+ cells. There was an average of 2 for *TERC* and *TERT* genes per cell (Supplementary Fig. 1S). Only in a few clinical cases, we observed that the average number of copies of the *TERT* gene was over 2 (in three CML-CP patients and in one CML-BP patient). Comparative analysis of expression profiles of *TERC* and *TERT* in two groups of patients showed a statistically significant increase only in the expression of *TERT* in the CML-BP group of patients (Fig. 2a–b). However, it should be noted that the level of *TERT* gene expression was at a very low level, undetectable in many samples, with a tendency to increase in CML-BP.

Next, we checked the transcriptional activity of the *DKC1* gene—nucleolar protein, which is responsible for maintaining *TERC* stability by interacting with H/ACA consensus sequence in *TERC*. The conducted comparison between the expression of the *DKC1* gene in two groups of CML CD34+ showed a statistically significant increase (2.1-fold) of *DKC1* expression in CML-BP cells in comparison with CML-CP cells (Fig. 2c). Additional correlation analysis conducted on CML CD34+ cells showed a statistically significant correlation between *DKC1* expression and the *BCR::ABL1* gene (*r* = 0.68 *p* < 0.0001) (Fig. 2g), and, interestingly, such significance was not found for *TERC* or *TERT* genes (Fig. 2e, f). Moreover, there was no significant difference in telomerase activity between CML-CP and CML-BC cells (Fig. 2d).

### The shelterin gene expression correlates with the level of *BCR::ABL1* in CML cells

**Fig. 3:**
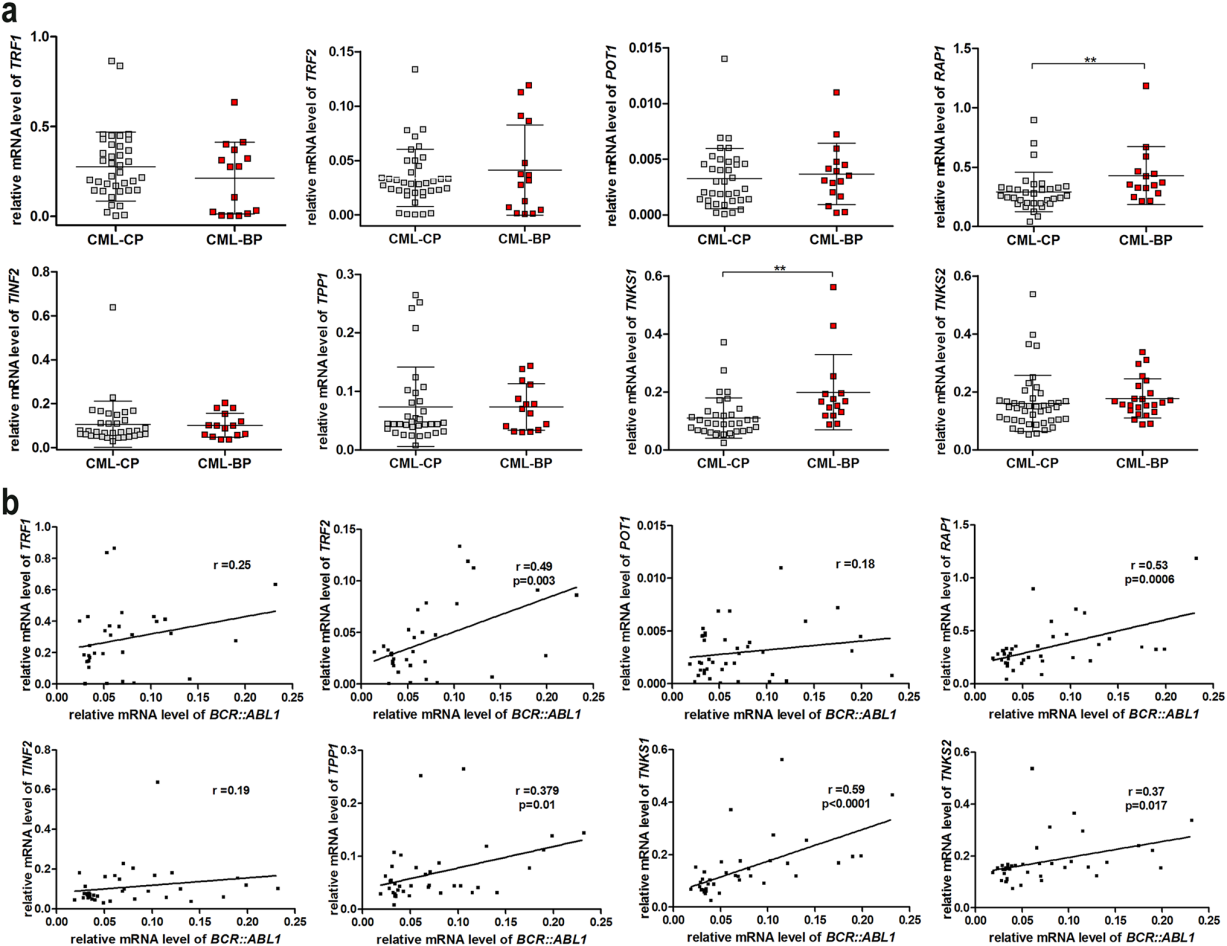
CML phase and *BCR::ABL1* expression-mediated changes in the expression of *TRF1* (*n* = 52), *TRF2* (*n* = 53), *POT1* (*n* = 52), *RAP1* (*n* = 48), *TINF2* (*n* = 49), *TPP1* (*n* = 49), *TNKS1* (*n* = 51) and *TNKS2* (*n* = 49) genes. **a** Comparison of *RAP1, POT1, TRF1, TRF2, TPP1, TNKS1* and *TNKS2* gene expression between CML-CP and CML-BP patients. The gene expression was normalized to *B2M* and *GUSB* and measured with qPCR, **p < 0.01 (non-parametric student *t* test and Mann–Whitney post-test). **b** Correlation between *BCR::ABL1* and *TRF1*, *TRF2*, *POT*, *RAP1*, *TINF2*, *TPP1*, *TNKS1* or *TNKS2* expression levels (Pearson’s (*r*), *p* < 0.05)

Comparative analysis of the expression profile of genes of the telomere complex, such as *TRF1, TRF2, POT1, RAP1, TTP1, TNKS1*, and *TNKS2* in CML-CP and CML-BP cells, showed a statistically significant increase in the expression of two genes: *TNKS1* and *RAP1* in CML-BP cells (*p* < 0.01) (Fig. 3a). The average level of *RAP1* expression was 1.4-fold higher for CML-BP cells and 1.72-fold higher for *TKNS1* in relation to CML-CP cells, respectively. A comparison between the expression of different genes of the shelterin complex and the level of *BCR::ABL1* transcription showed statistically significant correlations with the following genes: *RAP1* (*p* = 0.0006), *TRF2* (*p* = 0.003), *TPP1* (*p* = 0.01), *TNKS1* (*p* < 0.0001) and *TNKS2* (*p* = 0.017) (Fig. 3b).

## Discussion

During CML progression, the loss of genetic stability is manifested not only by a general increase in point mutations, DNA breakages, oxidative DNA damage, or disturbances of the epigenome, but also by changes in telomeric sequences (Boultwood et al. 1999; de Oliveira et al. 2022). The analysis of telomere length in hematopoietic (HSCs) and LSCs from the same patient shows that the average length of telomere in LSCs is much shorter, and this shortening correlates with the leukemic clone size (Bouillon et al. 2018). Moreover, according to the Hasford score, high-risk patients at diagnosis, reveal significantly greater telomere shortening rate compared to low-risk score patients, while intermediate-risk score patients exhibit an intermediate telomere shortening rate (Drummond et al. 2004). The obtained results confirm that telomeres were significantly shorter in CML-BP cells compared to cells from patients in CML-CP (Drummond et al. 2004). Thus, telomere shortening can be considered as a novel prognosis marker complementary to already established markers (Wang et al. 2014). The correlation analysis of the telomere length and the level of *BCR::ABL1* expression suggests that *BCR::ABL1* may induce dynamic changes in telomere length. Moreover, telomere shortening may contribute to senescence-associated inflammation and in turn disease progression in CML (Braig et al. 2014). CML cells might present nonrandom individual telomere length changes, such as shortening with different shorting rates and lengthening of telomeres located at some specific chromosome ends (Samassekou et al. 2009). It has been shown that the dynamics of individual telomere lengths might lead to telomere position effects, and in consequence inappropriate gene expression at subtelomeric regions (Koering et al. 2002). The analysis of the relationship between age of CML patients and mean length of telomeres has shown that telomere length slightly decreased with age. However, generally telomere length in CML-BP cells was still shorter than in CML-CP cells, so the age-related changes in telomere length during CML  do not seem to be the major factor responsible for telomere shortening.

In cancer cells, the lengthening of telomere sequences takes place through the reactivation of telomerase enzyme activity or alternatively through the process of recombination (Okamoto and Seimiya 2019)**.** The decreasing effectiveness of these mechanisms leads to excessive shortening of telomeres, resulting in chromosome instability, which often leads to cellular heterogeneity related to defective mechanisms of apoptosis (Murnane et al. 2012). Furthermore, chromosome changes can promote the production of many factors that work mainly locally, for instance the cytokines or growth factors, which may lead to inflammation or tumor growth (Andriani et al. 2016). Thus, understanding the mechanisms that regulate the length of telomeres may shed light on the processes of cell selection and adaptation that occur during the development of cancer, including CML. For these purposes, it would be interesting to find out the molecular factors that control the activity of telomerase. Telomerase, as a ribonucleoprotein enzyme complex, is composed of a subunit of reverse transcriptase (TERT) and an RNA component (TERC). The activity of human telomerase is controlled on three levels, namely, on the level of transcription, the assembly of subunits into an active enzyme, as well as of direct interaction of telomerase with proteins from the telomere complex.

Our comprehensive analysis of the activity of telomerase in CML CD34+ cells does not confirm earlier observations, which pointed to changes in the activity of this enzyme depending on the phase of disease in leukocyte cells of patients with CML (Ohyashiki et al. 1997). However, it should be noted that in the aforementioned work, the authors analyzed unfractionated cells from 33 CML-CP patients and 21 CML-BP patients. The cells at CML-BP exhibited a significant increase in telomerase activity (TA) (*p* = 0.016) and, at the same time, a statistically significant decrease in telomere length from 6.13 ± 1.68 kb in CML-CP to 4.53 ± 0.72 kb in CML-BP at *p* = 0.0005). The authors did not correlate their results with the level of expression or activity of BCR::ABL1 kinase. Drummond et al. arrived at similar conclusions, showing a lack of overexpression of *TERT* and lowered levels of *TERC* expression in CD34 + cells of CML-CP patients as compared with healthy subjects (Drummond et al. 2005). They postulated that the observed increase in TA in peripheral blood cells of patients with CML may be related to a heightened proportion of cells released from bone marrow into the periphery, rather than a true increase in intracellular telomerase activity.

Nevertheless, the association of telomerase upregulation with CML progression has been reported (Keller et al. 2009). Based on these results, telomere length, at least in the context of intact cell cycle checkpoints, could represent a valuable prognostic and/or predictive biomarker for disease progression, response to TKIs, and potentially for maintenance of response upon cessation of TKI treatment.

The analysis of expression profiles of *TERC* and *TERT* in two groups of patients showed a statistically significant increase only in the expression of *TERT* in the CML-BP group of patients. Thereby, the obtained results do not confirm earlier observations, which point to lowered expression of *TERT* along with the progression of CML (Campbell et al. 2006). Campbell et al., comparing the gene expression in the CML CD34+ cells isolated from 22 CML patients’ samples to the normal CD34+ cells, showed that expression of *TERT* was downregulated in over half of the samples from patients in the chronic phase, significantly downregulated in two out of three patients in the accelerated phase and in all CML CD34+ cells isolated from patients in blastic phase. The same authors also postulated that lowered transcription of *TERT* in the CML-BP stage is associated with the levels of *C-MYC*, the expression of which decreased as the disease progresses. Due to these divergences, extended research in this area is required. Nevertheless, our results show that the level of expression and number of copies of *TERT* cannot be considered as the main cause of changes in telomere length during progression. In this context, we checked the transcriptional activity of the *DKC1* gene–nucleolar protein, which is responsible for maintaining *TERC* stability. The role of *DKC1* in the progression and development of hematopoietic and solid tumors has been already described i.e., *DKC1* dysfunction leads to diminished TERC levels, a decrease in telomerase activity, and premature telomere shortening in males (Montanaro et al. 2010; Hirvonen et al. 2019). The conducted comparison between the expression of the *DKC1* gene in two groups of CML CD34+ showed a significant increase (2.1-fold) of *DKC1* expression in CML-BP cells in comparison with CML-CP cells. This result is in contrast with the observed decrease in the length of telomeres in CML-BP. This may suggest that *DKC1* overexpression in CML cells is not related to telomerase activity. A likely explanation for this biological phenomenon is the increase of CML cancer cells’ demand for DKC1 due to its role in post-transcriptional modification of rRNA necessary for the maintenance of an effective process of translation (Ge et al. 2010; Jack et al. 2011).

Comparative analysis of the expression profile of genes of the telomere complex showed a significant increase in the expression of two genes: *TNKS1* and *RAP1* in CML-BP cells. Campbell et al. 2006 previously showed that the expression of telomeric-associated proteins TEP1, TRF1, TRF2, TNKS1, and PinX1 was elevated in the majority of CML-CP and CML-AP patients and decreased during disease progression, with the exception of TEP1 (Campbell et al. 2006). However, it ought to be noted that the analysis of the expression of the genes studied had not been correlated with the expression of *BCR::ABL1* in individual samples, and the analysis was performed on one reference gene (*B2M*), which may have an impact on the obtained results, while our results were normalized to *B2M* and *GUSB.*

Moreover, contrary to the other researchers we have shown a positive correlation between increased expression of *TRF2*, *RAP1*, *TTP1*, *TNKS1*, and *TNKS2* genes and the level of expression of *BCR::ABL1*, and also simultaneously with a decrease in the length of telomeres. Nevertheless, one ought to remember that an increase in the expression of RAP1 observed here may not be related to changes in telomeres and could be merely another form of adaptation of CML cells to increased metabolic activity characteristic for cancer cells (Deregowska and Wnuk 2021). It is well known that RAP1 is a pleiotropic protein that is responsible for the regulation of cell metabolism, the production of conditions associated with inflammation, response to oxidative stress (Cai et al. 2017) and regulation of hematopoietic stem cell survival (Khattar et al. 2019). Therefore, due to the observed correlation between increased expression of *BCR::ABL1* and levels of *TRF2* expression, an alternative explanation may also be found in the following scenario: the increase of expression of *BCR::ABL1* during the progression of CML leads to an increase in levels of shelterin complex proteins, including the overexpression of *TRF1* and *TRF2*, which are known to be negative regulators of telomere length, and whose binding to telomeres is dependent on posttranslational modification of the poly-ADP-ribosylate by tankyrases 1 and 2 (van Steensel et al. 1998; Smogorzewska et al. 2000; Smogorzewska and de Lange 2004). Furthermore, overexpression of *TRF1* and *TRF2* may promote the nucleolytic activity of XPF on chromosome endings, leading to acceleration of telomere shortening (Muñoz et al. 2005).

## Conclusion

In summary, we show that the telomere length dynamics in CML cells including CD34+ cells is strictly related to the level of expression of *BCR::ABL1*, which stimulates an increase in expression of some shelterin genes, including *TRF2*, *RAP1* and *TPP1,* as well as other telomere-associated proteins: *DKC1*, *TNKS1*, and *TNKS2*, promoting the shortening of telomeres regardless of telomerase activity. Our results may help in better understanding of the mechanisms responsible for the loss of genome stability in CD34+ cells and CML progression. We believe that our results may also help in the future to find new therapeutic targets in leukemic stem cells and the development of effective therapy especially for advanced phases of the disease, but also may be helpful in other hematological malignancies.

## Supplementary Information

Below is the link to the electronic supplementary material.

Supplementary file1 (DOCX 351 KB)

## Data Availability

The datasets generated during and/or analyzed during the current study are available from the corresponding author on reasonable request.
