# Supplementary material for: The interplay between telomeric complex members and BCR::ABL1 oncogenic tyrosine kinase in the maintenance of telomere length in chronic myeloid leukemia
Source: J Cancer Res Clin Oncol. 2023 Mar 5;149(10):7103–12. doi: 10.1007/s00432-023-04662-w (PMC10374722; doi:10.1007/s00432-023-04662-w)
Supplement: Supplementary file 1 — Supplementary file1 (DOCX 351 KB) [file 432_2023_4662_MOESM1_ESM.docx]

*Supplementary Table 1S*. Primer sequences and UPL probe numbers (Roche)/probe sequences used for qRT-PCR

| **gene** | **primers [5’→ 3’]** | **UPL probe no/**  **Sequence [5’→ 3’]** |
| --- | --- | --- |
| *TRF1* | **F:** TGCTAAGTGAAAAATCATCAACCTT | #8 |
|  | **R:** TTGTTCTTGTCCTTTTGCTTTCT |  |
| *TRF2* | **F:** CCCACCGTTCTCAACCAA | #80 |
|  | **R:** GTTCCACTTGCCTTTGGGTA |  |
| *POT1* | **F:**TTATACGAGGTAGAAAGATGTCAACAG | #55 |
|  | **R:** GTTTCAAAATGGCACATAGTGG |  |
| *RAP1* | **F:** GGGCCAGGAGCATAAGTACC | #52 |
|  | **R:** GGAGTTCTCTTATTCTGTGGTTCC |  |
| *TPP1* | **F:** CTCTGTGCTCGGGTCCAA | #69 |
|  | **R:** GCATCCATCAGAAAGTGCAA |  |
| *TINF2* | **F:** GTCAGAGGCTCCTGTGGATT | #71 |
|  | **R:** TCCAGCTGACACAAGTACTCAAA |  |
| *TNKS1* | **F:** ATCAGGGAACGATTTTGCTG | #50 |
|  | **R:** CCACCATCTCTGTGTTCTCG |  |
| *TNKS2* | **F:** AATGTACAGCAACTCCTCCAAGA | #34 |
|  | **R:** CTTTGCAGCTTCCAGCAAT |  |
| *DKC1* | **F:** CCAGCGACCCCCACTTAT | #34 |
|  | **R:** CCTCACAACTCACCCAAAAGA |  |
| *TERC* | **F:** CGAGGTTCAGGCCTTTCA | #4 |
|  | **R:** CCACAGCTCAGGGAATCG |  |
| *TERT* | **F:** GCCTTCAAGAGCCACGTC | #19 |
|  | **R:** CCACGAACTGTCGCATGT |  |
| *BCR::ABL1* | **F:** TCCGCTGACCATCAATAAGGA | CCCTTCAGCGGCCAGTAGCATCTGA |
|  | **R:** CACTCAGACCCTGAGGCTCAA |  |
| *B2M* | **F:** GAGTATGCCTGCCGTGTG | CCTCCATGATGCTGCTTACATGTCTC |
|  | **R:** AATCCAAATGCGGCATCT |  |
| *GUSB* | **F:** GAAAATATGTGGTTGGAGAGCTCATT | CCAGCACTCTCGTCGGTGACTGTTCA |
|  | **R:** CCGAGTGAAGATCCCCTTTTTA |  |

**Supplementary Figure 1S**


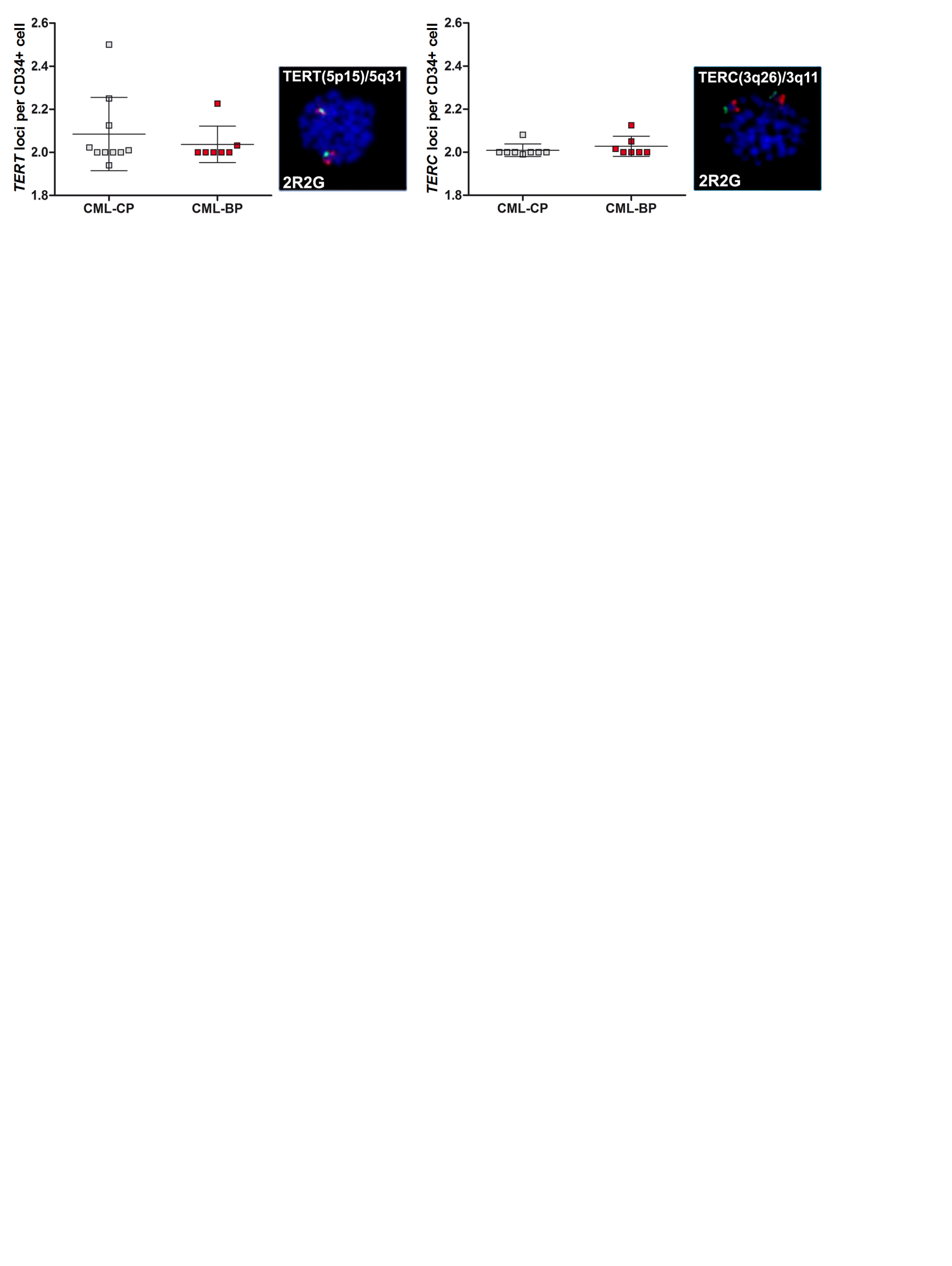


Supplementary Fig. 1S *BCR::ABL1*-mediated effect on *TERC, TERT* gene copy number variation: The mean number of *TERC* signals per cell. n=15. Representative metaphase microphotographs with typical signals localized to two chr3p26 (signals pattern 2R2G). The *TERC* probe is direct-labeled with PlatinumBright™550 (red) while control region (3q11) is direct-labeled with PlatinumBright™495 (green). The mean number of *TERT* signals per cell. n=17. Representative metaphase microphotographs with signals localized to two chr5p15 (signals pattern 2R2G). The *TERT* probe is direct-labeled with PlatinumBright™550 (red) while control region (5q31) is direct-labeled with PlatinumBright™495 (green). Nuclei were counterstained by DAPI.
